# Adjuvant therapy in the treatment of gallbladder cancer: a meta-analysis

**DOI:** 10.1186/s12885-015-1617-y

**Published:** 2015-09-03

**Authors:** Ning Ma, Hui Cheng, Baodong Qin, Renqian Zhong, Bin Wang

**Affiliations:** 1 Department of Laboratory Diagnostics, Changzheng Hospital, Second Military Medical University, 415 Fengyang Road, Shanghai, 200041 China; 2 Clinical Laboratory, 85th Hospital of PLA, 1328 Huashan Road, Shanghai, 200052 China; 3 Department of Hematology, Changhai Hospital, the Second Military Medical University, Shanghai, 200433 China; 4 Department of Oncology, Changhai Hospital, Second Military Medical University, 168 Changhai Road, Shanghai, 200433 China

## Abstract

**Background:**

The benefit of adjuvant therapy (AT) for gallbladder cancer (GBC) is unclear as evidenced by conflicting results from nonrandomized studies. Here we aimed to perform a meta-analysis to determine the impact of AT on overall survival (OS).

**Methods:**

We used data from MEDLINE, EMBASE and the Cochrane Collaboration Library and published between October 1967 and October 2014. Studies that evaluated AT compared with curative-intent surgery alone for resected GBC were included. Subgroup analyses of benefit based on node status, margins status, and American Joint Committee on Cancer (AJCC) staging were prespecified. Data were weighted and pooled using random-effect modeling.

**Results:**

Ten retrospective studies involving 3,191 patients were analyzed. There was a nonsignificant improvement in OS with AT compared with surgery alone (hazard ratio [HR], 0.76; 95 % confidence interval [CI], 0.56–1.03). A significant improvement was observed in OS with chemotherapy (CT) compared with surgery alone (HR, 0.42; 95 % CI, 0.22–0.80) by sensitivity analysis. The greatest benefit for AT was also observed in those with R1 disease (HR, 0.33; 95 % CI, 0.19–0.59), LN-positive disease (HR, 0.71; 95 % CI, 0.63–0.81), and AJCC staging meeting or exceeding tumor Stage II (HR, 0.45; 95 % CI, 0.26–0.79), but not in those with LN-negative or R0 disease.

**Conclusion:**

Our results strongly support the use of CT as an AT in GBC. Moreover, patients with node positivity, margin positivity, or non-stage I disease are more likely to benefit from AT.

## Background

Gallbladder cancer (GBC) is an uncommon but the most aggressive biliary tree cancer (BTC). To date, complete surgical resection offers the only chance for cure. Worldwide, GBC is the sixth most common gastrointestinal cancer with an annual incidence rate of 2.2 per 100,000 [1, 2]. In the United States, GBC accounts for approximately 9,760 new cases and 3,370 new deaths per year [3]. However, only 10 % of patients who present with early-stage GBC are considered surgical candidates.

A recent study by Valle J et al. showed that longer overall survival (OS) with gemcitabine in combination with cisplatin than with gemcitabine alone in patients with advanced or metastatic BTC [4]. However, established adjuvant treatments (AT) for GBC are lacking and much debate remains about whether AT affects survival in GBC. Regarding AT for GBC, only one phase III multicenter prospective randomized controlled trial (RCT) indicated that patients with gallbladder carcinoma who undergo R1 but not R0 resections may derive some benefit from systemic chemotherapy [5]. However, other trials that had examined the values of AT, including chemotherapy (CT), radiotherapy (RT), and chemoradiotherapy (CRT), were limited by their small numbers of patients in their retrospective and non-randomized study design.

There are currently no meta-analyses of AT for GBC on the basis of retrospective data. As such, the aim of this study was to conduct a meta-analysis to identify whether AT, i.e., RT, CT, or CRT, could improve OS compared with surgery alone for the entire group or subgroups (node status, margins status, American Joint Committee on Cancer [AJCC] staging, and countries vary) of GBC on the basis of those retrospective data.

## Methods

### Data collection

An electronic search of the MEDLINE, EMBASE and the Cochrane Collaboration Library were performed using Internet explorer 10. Searches were limited to human studies and English-language publications. The main keywords used for the search were “gallbladder cancer” and “adjuvant therapy”. The published years were limited to 1976–2014. A MeSH term search was performed in MEDLINE. Citation lists of retrieved articles were manually screened to ensure search sensitivity. We downloaded the available studies from those databases or contacted with authors if needed.

### Study selection

**Fig. 1:**
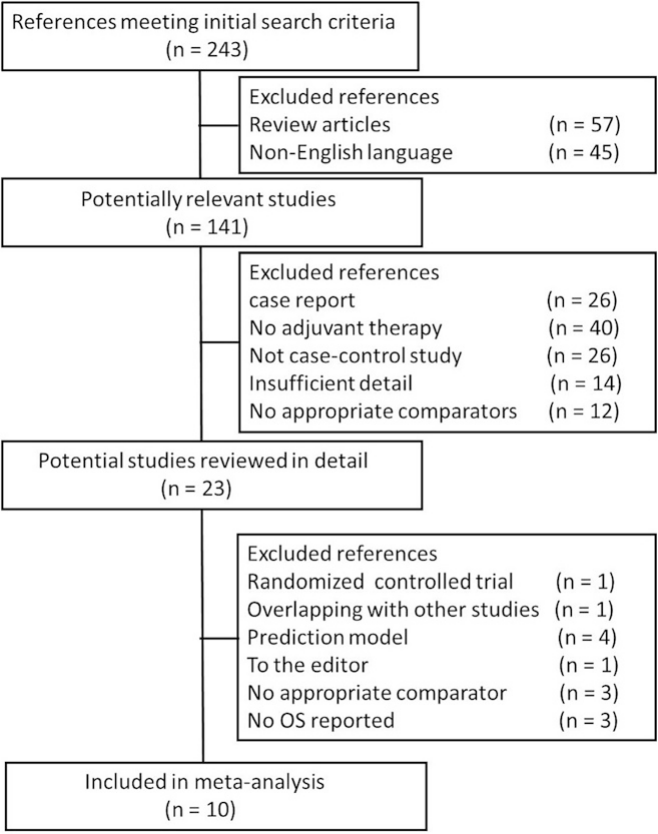
Flow chart showing the progress of trials through the review

The relevant clinical trials were manually selected carefully based on the following criteria: (1) case–control design of non-randomized study; (2) patients diagnosed with GBC according to histopathological or cytological evidence; (3) patients underwent AT defined as CT, RT, or both administered after curative-intent surgery, and patients who underwent curative-intent surgery alone as a comparator group should be included in those studies; (4) information collected including hazard ratio (HR) for OS along with 95 % confidence interval (CI) or relevant data. When searched references referred to the same studies, the more recently published and larger studies were included. We also defined curative-intent resections as no gross disease remaining (i.e., negative margins [R0] or microscopic positive margins [R1]), thus excluding macroscopic involvement (R2) resections [6]. The procedure of inclusion and exclusion criteria of the evaluated studies was listed in Fig. 1.

### Data extraction

Three investigators (Ning Ma,Hui Cheng and Baodong Qin) searched the publications independently using standardized data abstraction forms. When the three investigators discovered different results, an independent expert in oncology made the final decision. Details such as first author, year of publication, patient characteristics, institution, country of study, and patient number must be included in these publications. T stage, AJCC stage, and nodal and resection margin statuses were collected. Details on therapeutic interventions, including surgical procedure, CT regimen, radiation type and dosage, and treatment schedule were also collected.

The details of response rate, median/overall survival, HR for OS (HROS) and their 95 % CI, and adverse events must be collected as outcomes from these studies. If HR and 95 % CI were not given, we estimated them as described below depending on the data provided in the publication. The estimated HR and its standard error was obtained from the report results or calculated using two of the following parameters: the O - E statistic (difference between numbers of observed and expected events), the CI for the HR, and the log-rank statistic or its P value. If these were not available, the total numbers of events, number of patients at risk in each group, and log rank statistic or its P value were used to allow for an approximation of the HR estimate [7–9]. In addition, Kaplan-Meier curve was used to calculate HR and its standard error to verify those results calculated above. First of all, we divided Kaplan-Meier plot schematically into time intervals to obtain the data of survival rates of event-free on research and control groups. The data of HR, V and O-E then could be obtained according to the method provided by Tierney JF et al. [10]. The estimated HR and its standard error could be obtained according the method mentioned above, and be verified with the data obtained above. If this kind of method was used, three independent persons read the curves to reduce the inaccuracy in the extracted survival rates.

### Statistical analysis

The relative frequencies of survival between AT and curative-intent surgery alone were expressed as HR and their 95 % CI. Statistical heterogeneity was tested and a random effect model was applied at last in calculating the overall HR. The pooled HR for OS was calculated then. As for key components of design, rather than quality scores themselves, may be more important [11], subgroup and sensitivity analyses were was designed in our meta-analysis to identify whether AT could improve OS compared with surgery alone.

Subgroup analyses were conducted that included node-positive/negative, margin-positive (R1)/negative (R0) disease, and treatment consisting of CT, RT, or CRT. Few studies were conducted solely in these populations. Thus, studies in which ≥50 % of the patients had nodal -positive/negative, R0/R1 disease on pathology and ≥50 % of the patients meeting or exceeding tumor stage II were calculated as the subgroup analysis. All of the analyses were performed using STATA 11.0. This meta-analysis of the observational studies was written according to the MOOSE group [11].

## Results

### Study characteristics

A total of 243 studies met the initial search criteria, and we identified 11 studies including one RCT [5] and 10 retrospective studies [12–21]. Those 10 retrospective studies were identified as eligible for inclusion in the pooled analysis (Fig. 1). These studies incorporated 3,191 patients in which 2,375 were treated with surgery alone (Lap choly only, Conversion open choly, Radial second resection, Primary open choly, or Hemihepatectomy) and 816 received AT. The details of HR for OS (HROS) and their 95 % CI were obtained from these studies. The types of AT,type and duration of chemo, radiation dosing, and other clinical data of those studies were collected and listed in Table 1.

**Table 1:** Characteristics of the included studies

Author	Study period	Institution/Country	No. of patients		Adjuvant therapy		Outcome	Margin positive		Margin negative		Node positive		Node negative		Stage ≥ II	
			Treatment	Control	Therapy	Regimen (details)		Treatment	Control	Treatment	Control	Treatment	Control	Treatment	Control	Treatment	Control
Lee [12]	1994–2011	Korea	135	83	NSR	FU/GEM(NR) + RT(NR)	OS	<23 %	<37 %	>77 %	>63 %	<27 %	<43 %	>73 %	>57 %	NR	NR
	Subgroup (CT)		73	83	CT	FU/GEM(NR)	OS	NR	NR	NR	NR	NR	NR	NR	NR	NR	NR
	Subgroup (CRT)		62	83	CRT	FU/GEM + RT (NR)	OS	NR	NR	NR	NR	NR	NR	NR	NR	NR	NR
Murakami [13]	1990 to 2010	Japan	11	51	CT	GEM + S-1	OS	NR	NR			NR	NR	NR	NR	NR	NR
	Subgroup (stage II/III)		10	31	CT	(10 cycles every 2 weeks with GEM 700 mg/m^2^ on day 1 and S-l 50 mg/m^2^ for 7 consecutive days)	OS	NR	NR			NR	NR	NR	NR	100 %	100 %
Gold [14]	1985 to 2004	United States	25	48	CRT	FU + RT (median dosage 50.4 Gy (range, 19.75–54.0) in 28 fractions and concurrent 5-FU given as an interrupted bolus of 500 mg/m^2^ for 3 successive days during Week 1 of RT and repeated during Week 5)	OS	0	0	100 %	100 %	56 %	13 %	32 %	67 %	80 %	21 %
Liang [15]	1980 to 2005	China	62	88	NSR	FU/CF/Adr/Dox/Mi t/Cisp/RT (CT:NR and RT:range 12–66 Gy; mean 51.07 Gy)	OS	NR	NR			NR	NR	NR	NR	76 %	92 %
Duffy [16]	1995 to 2005	United States	24	99	NSR	GEM/FU/GEM + Cape + RT(NR)	OS	0	0	100 %	100 %	NR	NR	NR	NR	NR	NR
					CT in 8 PTS, CRT in 16 PTS												
Mojica [17]	1992 to 2002	United State (SEER)	424	1901	RT	NR	OS	NR	NR	NR	NR	30 %	15 %	NR	NR	79 %	51 %
	Subgroup (Node Positive)		127	277	RT	RT(NR)	OS	NR	NR	NR	NR	100 %	100 %	NR	NR	NR	NR
	Subgroup (T3N0)		71	218	RT	RT (NR)	OS	NR	NR	NR	NR	NR	NR	100 %	100 %	NR	NR
	Subgroup (T1-2 N0)		115	708	RT	RT (NR)	OS	NR	NR	NR	NR	NR	NR	100 %	100 %	NR	NR
Balacbandran [18]	1989 to 2000	India	73	44	CRT	CRT (NR)	OS	NR	NR			31 %	25 %	13 %	5 %	89 %	86 %
Lindell [19]	1991 to 1999	Sweden	10	10	RT	IORT + EBRT	OS	50 %	50 %	NR	NR	30 %	20 %	70 %	80 %	80 %	100 %
						I0RT(20Gy) + EBRT(40Gy, 20 fraction, 5 days a week druing 6 weeks)											
Itoh [20]	1994 to 2004	Australia	5	13	RT	EBRT (total dose of 45. 2 Gy (range, 45. 0–56. 7) for 2–6 weeks, using a fraction size of 1. 8–2. 0 Gy)	OS	60 %	31 %	40 %	69 %	NR	NR	NR	NR	NR	NR
Todoroki [21]	1976 to 1996	Japan	47	38	RT	IORT + PORT	OS	59 %	50 %	NR	NR	NR	NR	NR	NR	100 %	100 %
	Subgroup(Rl)		28	19	RT	(I0RT(21 ± 0.5Gy, ranging from 15–30 Gy) + P0RT(40 ± 1.9 Gy, ranging from 24.8-54Gy, using a fraction size 1. 8–2.0 Gy))	OS	100 %	100 %	NR	NR	NR	NR	NR	NR	NR	NR

Abbreviations: *OS* overall survival, *CT* chemotherapy, *RT* radiation, *CRT* chemoradiotherapy, *NSR* non-single regimen (perhaps including CT, RT, and CRT), *EBRT* external beam radiation therapy, *FU* fluorouracil, *MMC* mitomycin C, *NR* detail not reported, *FU* 5-fluorouracil, *IORT* intraoperative radiotherapy, *EBRT* external beam radiotherapy, *GEM* gemcitabine, *Cape* capecitabine, *Cisp* cisplatin, *Mit* mitomycin-C, *Dox* doxorubicinol, *ADR* Adriamycin, CF leucovorin, *SEER* Surveillance, Epidemiology, and End Results, *NA* not applicable, *PTS* patients

### Meta-analysis

**Fig. 2:**
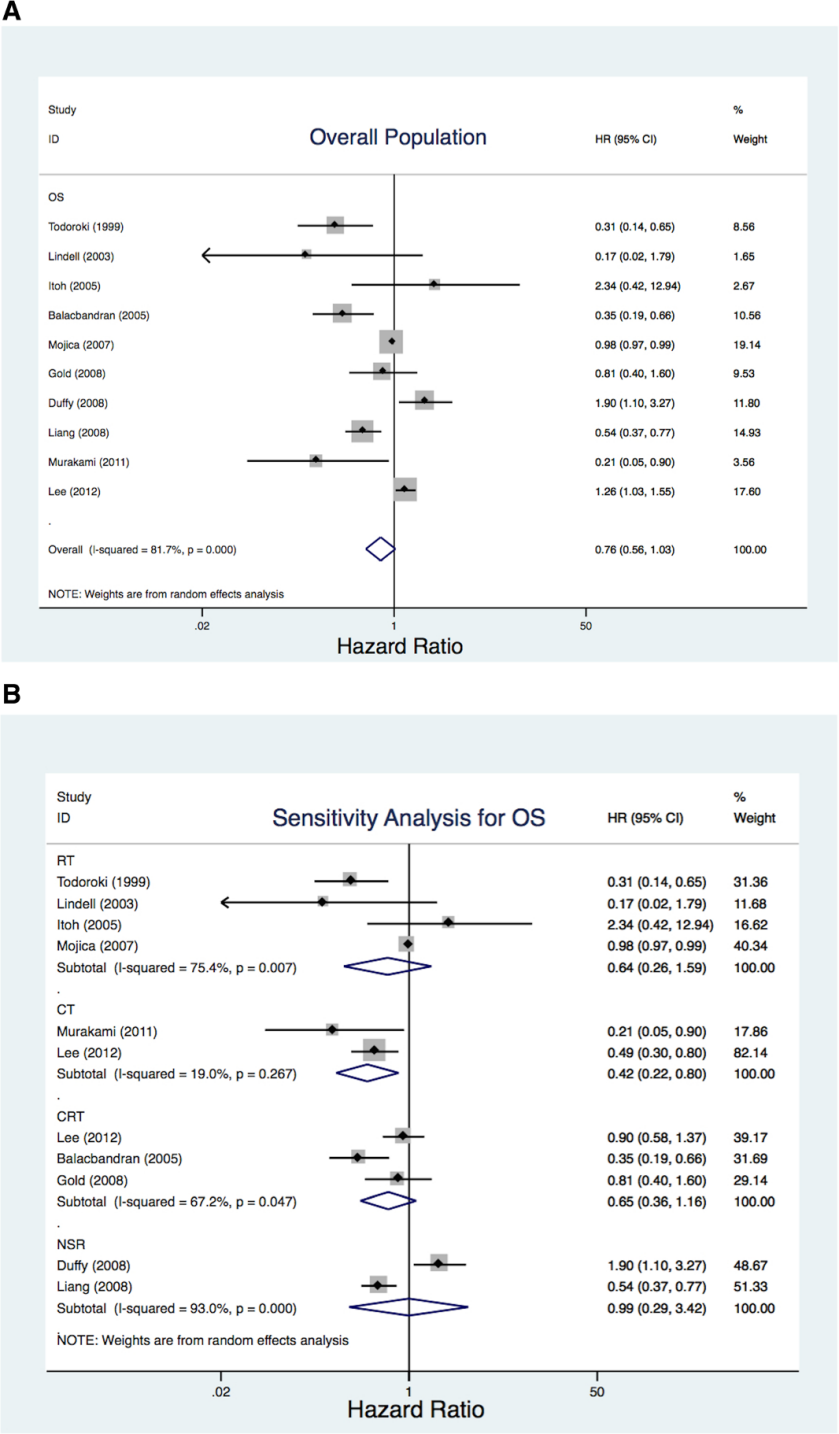
Efficacy outcomes for overall population and sensitivity analysis. **a**. Overall population. **b**. Sensitivity analysis for overall survival

In calculating the overall HR, statistical heterogeneity was tested before and the value of p is 0.000. Random effect model was applied then and as a result, pooled data showed a nonsignificant improvement in OS with any AT compared with surgery alone (HR, 0.76; 95 % CI, 0.56–1.03; Fig. 2a) in the overall population.

Subgroup analysis showed a significant improvement in survival with CT compared with surgery alone (HR, 0.42; 95 % CI, 0.22–0.80) but not statistically significant compared to CRT (HR, 0.65; 95 % CI, 0.36–1.16) or RT (HR, 0.64; 95 % CI, 0.26–1.59; Fig. 2b).

### Heterogeneity and sensitivity analyses

#### Margin status

**Fig. 3:**
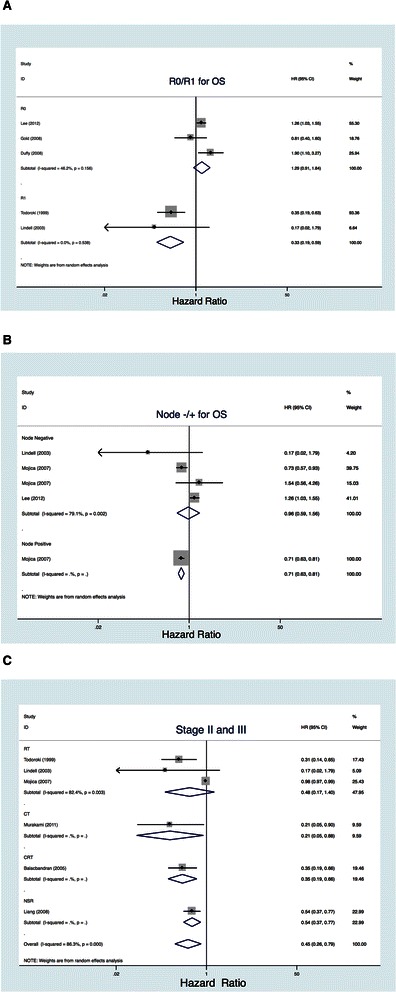
Efficacy outcomes for margin status and node status. **a**. R0/R1 for OS. **b**. Node −/+ for OS. **c**. Stages II and III

Two studies [19, 21] reporting margin positivity (R1) (*n* = 105) according to our prespecified definition (≥50 %) were analyzed independently [6]. Pooled data confirmed a significant benefit for AT in margin-positive patients (HR, 0.33; 95 % CI, 0.19–0.59; Fig. 3a).

Three studies reporting margin negative (R0) (*n* = 414) according to our prespecified definition (≥50 %) were also analyzed independently [12, 14, 15]. We found that patients with GBC and R0 resection could not benefit from AT compared with surgery alone (HROS, 1.29; 95 % CI, 0.91–1.84; Fig. 3a).

#### Node status

Three studies reporting nodal positive (*n* = 404) or negative (*n* = 1350) according to our prespecified definition (≥50 %) were analyzed independently [12, 17, 19]. Pooled data showed a significant benefit for any AT in node-positive disease (HR, 0.71; 95 % CI, 0.63–0.81; Fig. 3b) but no statistically significant benefit in node-negative disease (HR, 0.96; 95 % CI, 0.59–1.56; Fig. 3b).

#### AJCC staging

As mentioned above, all 11 studies were published between 1999 and 2012. The 6^th^ AJCC staging was adopted by most of these studies (AJCC Cancer Staging Manual, 2002) [22], so clinical disease staging was adopted according to the AJCC staging system (6th edition) to avoid stage migration.

**Fig. 4:**
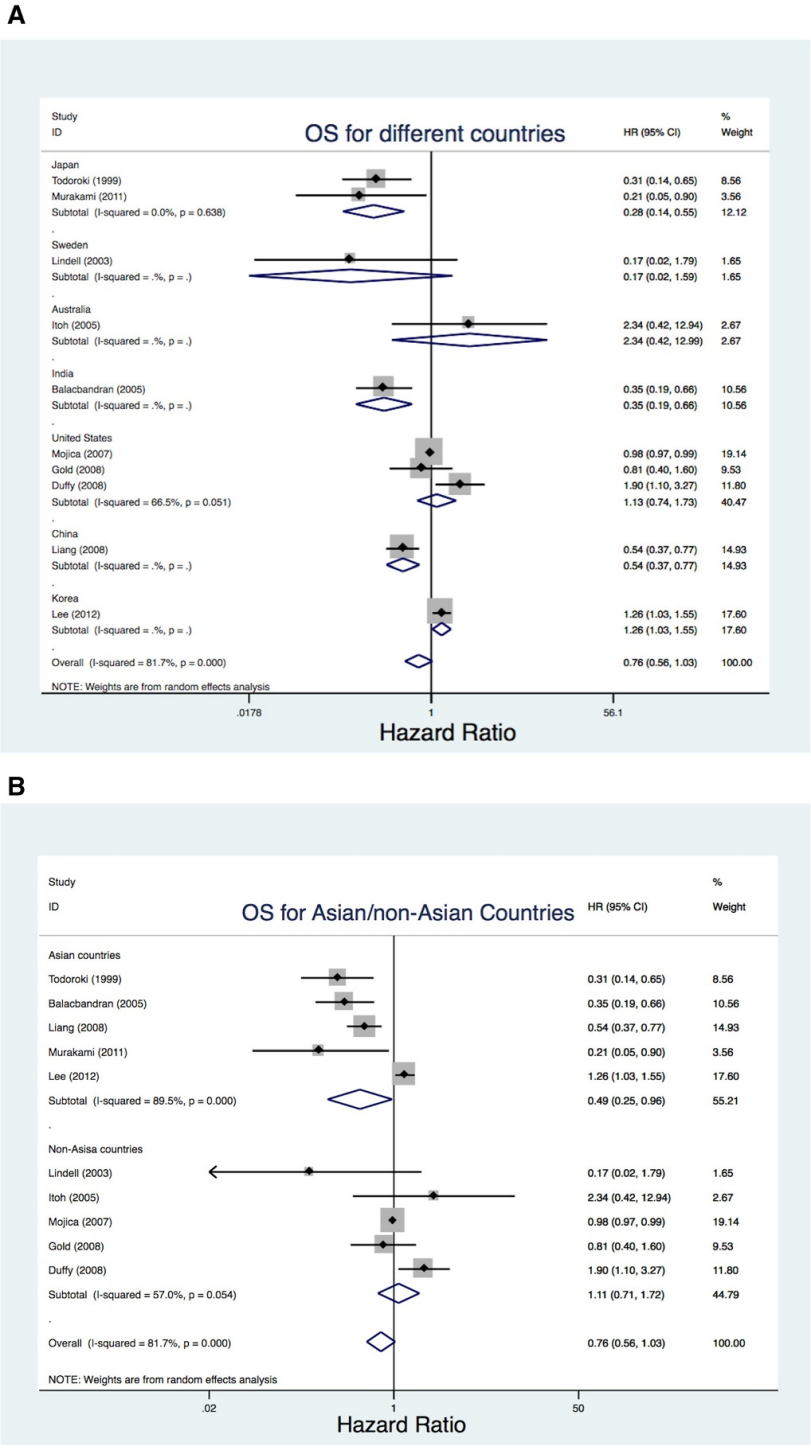
Efficacy outcomes for difference of country and cumulative meta-analysis over time. **a**. Different countries. **b**. Asian/non-Asian countries

AT were less adopted on GBC of Tumor, Node, Metastasis staging T1 N0M0/T2N0M0. As a result, the AJCC staging of most of patients in these 11 studies met or exceeded T2N1M0 or T3N0M0, which is stage II in the 6th AJCC staging system. Among these 11 studies, seven meeting or exceeding tumor stage II (*n* = 2,738) according to our prespecified definition (≥50 %) were analyzed independently [13, 15, 17–19, 21]. Pooled data confirmed a significant benefit for any AT in those patients (HR, 0.45; 95 % CI, 0.26–0.79; Fig. 4a). Subgroup analysis showed a significant improvement in survival with CT compared with surgery alone (HR, 0.21; 95 % CI, 0.05–0.88) but not with RT (HR, 0.48; 95 % CI, 0.17–1.40; Fig. 4a).

To further substantiate our findings, the studies with 100 % of the patients meeting or exceeding tumor stage II were analyzed independently. Two studies complied with this (*n* = 126) [13, 21]. As a result, the pooled data confirmed a significant benefit for any AT in these patients (HR, 0.28; 95 % CI, 0.14–0.56; figure not shown).

#### Results vary among countries

We also analyzed the pooled HR with CI by country. Our meta-analysis showed a significant improvement in OS with AT among Asian countries (HR, 0.49; 95 % CI, 0.25–0.96, Fig. 4a, b) but not among non-Asian countries (HR, 1.11; 95 % CI, 0.71–1.72, Fig. 4a, b).

### Evaluation of publication bias

**Fig. 5:**
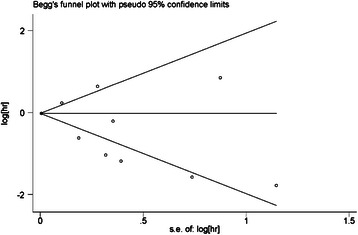
Begg’s funnel plot

Begg’s funnel plot and Egger’s test were performed to assess the publication bias of the literature. Evaluation of publication bias for AT versus surgery alone showed that both Begg’s and Egger’s test findings were not significant (*p* = 0.788 and 0.284) (Fig. 5). The meta-analysis was not dominated by any individual study, while removing any study at a time made no difference (data not shown). These results indicated no evidence of publication bias in our meta-analysis.

## Discussion

GBC is an uncommon cancer but the most aggressive BTC. Because of the lack of randomized data, there are no established post-resection AT for GBC [23, 24]. The aim of our study was to perform a meta-analysis to identify whether AT could improve OS.

It is well known that meta-analysis is mainly based on RCT. If there were insufficient RCT, a systemic assessment of non-RCT is needed. According to the Cochrane systematic review (http://www.cochrane.org/), non-RCT or retrospective studies may play a complementary role under these circumstances [11].

The meta-analysis by Horgan et al. recently published in *Journal of Clinical Oncology* reported a nonsignificant improvement in OS with AT compared with surgery alone for BTC and the GBC subgroup [6, 25]. In that study, odds ratio (OR) was chosen as the effect label instead of HR. What is more, only four studies including one RCT and three non-RCT were eligible for inclusion in that pooled analysis and their results were based on the study of RCT combined with retrospective and non-randomized studies. Just as the authors stated, OR is a less robust measure of survival because it does not consider survival duration prior to death. Contrary to their study, our meta-analysis was on the basis of retrospective data and HR instead of OR.

As such, we performed this meta-analysis of our 10 collected studies (involving 3,191 patients in 10 retrospective studies) to identify whether AT could improve OS compared with surgery alone using HR as the effect label following the methodology described by Parmar et al. [7, 9]. Before this, we excluded the studies that did not provide case–control design, adjuvant therapy, sufficient detail, or appropriate comparators. The studies of single case reports, RCT, and review were also excluded which mentioned above (Fig. 1). Our pooled analysis demonstrated a nonsignificant benefit in OS in unselected patients. Our subgroup analysis showed a significant improvement in survival with CT (HR, 0.42; 95 % CI, 0.22–0.80) compared with surgery alone but a nonsignificant improvement in survival with RT and CRT. However, this does not mean that RT and CRT could not play a positive role since their HR were 0.64 and 0.65, respectively (Fig. 2b).

Similarly with CT in OS, the sensitivity analyses indicated that post-resection AT seems beneficial in subgroups of high-risk patients, such as those with node and margin positivity, but not in patients with node negative or R0 disease (Fig. 3a, b). Sensitivity analyses also indicated the significant benefit of AT, especially CT, in patients with non-stage I disease (Fig. 3c).

We also conducted our meta-analysis based on nationality. Interestingly, our results showed a significant improvement in survival with AT in Asian countries but not in non-Asian countries (Fig. 5a, b). What could account for the difference? Could differences in race be a factor? These questions are worthy of future RCT.

The only available RCT showed that the use of adjuvant RT is associated with improved survival in patients with LN-positive (*P* < 0.0001) or stage IIa (T3N0M0) (*P* = 0.011) but not in patients with stage I disease [5]. Just as this study and Horgan [6] showed, our overall analysis also supports the use of AT for patients with LN-positive, R1, or AJCC stage > II GBC. It is known that there is a lack of randomized GBC data, so we performed this meta-analysis of observational studies without RCT according to the MOOSE group.

Our study has some limitations. In our Meta analysis, the quality of the studies included was various and the observational studies we included had much heterogeneity. Selection bias could distort the relationship between adjuvant therapy and overall survival. Therefore, we used random-effects modeling, made OS as the only end point and used sensitivity analyses (RT, CT, CRT, node status, margins status, AJCC stage, and multiple country analysis) to address this. As mentioned above, we calculated HR and 95 % CI using two of the following parameters: the O - E statistic, the CI for the HR, and the log-rank statistic or its P value. As we know, this kind of *estimated* value not the true value. To ensure the accuracy of the results, three investigators (Ning Ma,Hui Cheng and Baodong Qin) calculated HR and 95 % CI independently. Furthermore, Kaplan-Meier curve was also used if possible to calculate HR and 95 % CI to verify these results.

In addition,on the one hand, as most of the included studies were small sample case–control studies, the possibility of a type II error exists. On the other hand, the results of the non-RCT may be overstated. Begg’s funnel plot and Egger’s test were performed to assess the publication bias of the literature. As a result, these results indicated no evidence of publication bias in our meta-analysis.

## Conclusion

Our analysis provides reasonable support for the use of CT as an AT in patients with GBC. Moreover, patients with node positivity, margin positivity, or non-stage I disease are more likely to benefit from AT. We believe that the results of our meta-analysis will contribute to the use of CT as an AT in patients with GBC, especially those with the high risk factors described above. However our meta-analysis is based on the observational studies and not randomized controlled trial (RCT). Further research especially RCT is needed to better characterize the benefit of adjuvant therapy for gallbladder cancer.
